# Pre-Implementation Assessment of a Sexual Health eClinic in Canadian Oncology Care

**DOI:** 10.3390/curroncol32070395

**Published:** 2025-07-10

**Authors:** Taylor Incze, Dalia Peres, Steven Guirguis, Sarah E. Neil-Sztramko, Jackie Bender, Dean Elterman, Shabbir M. H. Alibhai, Antonio Finelli, Phil Vu Bach, Emily Belita, Gerald Brock, Julia Brown, Jeffrey Campbell, Trustin Domes, Andrew Feifer, Ryan Flannigan, Celestia Higano, Jesse Ory, Premal Patel, Monita Sundar, Luke Witherspoon, Andrew Matthew

**Affiliations:** 1 Faculty of Health Sciences, McMaster University, Hamilton, ON L8S L8S 4L8, Canada; inczet@mcmaster.ca (T.I.); neilszts@mcmaster.ca (S.E.N.-S.); belitae@mcmaster.ca (E.B.); 2 Department of Surgical Oncology, Princess Margaret Cancer Centre, University Health Network, Toronto, ON M5G 2C4, Canada; dalia.peres@uhn.ca (D.P.); steven.guirguis@uhn.ca (S.G.); antonio.finelli@uhn.ca (A.F.); 3 Department of Supportive Care, Princess Margaret Cancer Centre, University Health Network, Toronto, ON M5G 2C4, Canada; jackie.bender@uhn.ca (J.B.); shabbir.alibhai@uhn.ca (S.M.H.A.); 4 Division of Urology, Department of Surgery, University Health Network, University of Toronto, Toronto, ON M5T 2SB, Canada; dean.elterman@uhn.ca; 5 Division of Urology, Department of Surgery, University of Alberta, Edmonton, AB T6G 2R3, Canada; pbach@ualberta.ca; 6 Division of Urology, Department of Surgery, Western University, London, ON N6A 3K7, Canada; gerald.brock@sjhc.london.on.ca (G.B.); jeffrey.campbell@sjhc.london.on.ca (J.C.); 7 Royal Victoria Regional Health Centre, Barrie, ON L4M 6M2, Canada; brownj@rvh.on.ca; 8 Division of Urology, Department of Surgery, University of Saskatchewan, Saskatoon, SK S7N 5A5, Canada; trustin.domes@usask.ca; 9 Division of Urology, Department of Surgery, Trillium Health Partners, Toronto, ON M9C 1A5, Canada; andrew.feifer@thp.ca; 10 Prostate Cancer Supportive Care Program, Vancouver, BC V6T 1Z4, Canada; ryan.flannigan@ubc.ca (R.F.); thigano@u.washington.edu (C.H.); monita.sundar@vch.ca (M.S.); 11 Department of Urology, Dalhousie University, Halifax, NS B3H 4R2, Canada; jesse.ory@dal.ca; 12 Department of Surgery, University of Manitoba, Winnipeg, MB R3T 2N2, Canada; ppatel5@hsc.mb.ca; 13 Ottawa Hospital Research Institute, Ottawa, ON K1Y 4E9, Canada

**Keywords:** sexual dysfunction, oncosexology, prostate cancer, survivorship care, virtual health, sexual health rehabilitation, implementation science, CFIR

## Abstract

Sexual dysfunction is a prevalent and often under-addressed concern among prostate cancer survivors, significantly affecting quality of life for patients and their partners. The True North Sexual Health and Rehabilitation eClinic (SHAReClinic) is a virtual, biopsychosocial intervention developed to improve access to sexual health support for prostate cancer survivors and their partners. This study used a qualitative descriptive design to examine barriers and facilitators influencing the integration of SHAReClinic into oncology care across nine Canadian health care centres. Semi-structured interviews were conducted with 17 knowledge users, including health care providers and institutional leaders. Data were analyzed using a hybrid deductive–inductive thematic approach guided by the Consolidated Framework for Implementation Research (CFIR) 2.0. Participants described SHAReClinic as a much-needed resource, particularly in the absence of standardized sexual health pathways in oncology care. The virtual format was seen as accessible and well suited to addressing sensitive topics. However, limited funding, lack of institutional support, and workflow integration challenges emerged as primary barriers to implementation. Findings offer practical, theory-informed guidance for integrating SHAReClinic into oncology care and highlight key considerations for developing sustainable and scalable survivorship care models.

## Simple Summary

Sexual health concerns are common among people who have had prostate cancer, but many do not receive the support they need. The Sexual Health and Rehabilitation eClinic (SHAReClinic) is a virtual clinic designed to provide personalized sexual health education and counseling to patients and their partners. In this study, we interviewed health professionals and hospital leaders across Canada to understand what might help or prevent SHAReClinic from being successfully used in cancer care. Participants said the clinic filled an important need and appreciated that it was flexible, private, and easy to access. However, they also pointed out challenges such as limited funding and potential difficulties with fitting it into existing workflows. Based on this feedback, we developed practical recommendations to support future implementation. These insights will help guide the next streps in expanding sexual health services across cancer care settings.

## 1. Introduction

### 1.1. Sexual Dysfunction in Prostate Cancer

Sexual dysfunction (SD) is among the most prevalent and distressing side effects of prostate cancer (PCa) treatment, significantly affecting health-related quality of life for survivors and their partners [1,2,3,4]. Research on individuals post-radiation therapy and post-radical prostatectomy indicates that 24–60% and 20–90% experience SD, respectively [1,2]. One recent survey of men with PCa found 91% experienced erectile dysfunction and 98% developed new-onset, post-treatment sexual problems [5] Partners are also deeply affected, often reporting distress, disconnection, and strain in their relationships [4,6]. Despite these pervasive impacts, sexual health issues are frequently underreported and inadequately addressed in clinical settings [7,8]. PCa patients describe the sexual health care they do receive as fragmented, poorly timed, and impersonal, reinforcing the need for more accessible, patient-centred support [9].

### 1.2. True North Sexual Health and Rehabilitation eClinic (SHAReClinic)

Sexual health care remains an unmet need in oncology, with limited resources available to address patient concerns [7,10,11]. In alignment with Cancer Care Ontario Guidelines, the development of specialized sexual health clinics for oncology patients offers valuable benefits, including dedicated time to address sexual health concerns, access to expert knowledge, and comprehensive biopsychosocial care [12]. The biopsychosocial approach recognizes that sexual health is influenced not only by physical recovery but also by psychological well-being and social factors, such as relationships and partner support.

To address this gap, the Movember Foundation funded a pan-Canadian team of experts and patient/partner advocates to develop an innovative care model that responds to the sexual health concerns of PCa patients and their partners. This interdisciplinary collaboration integrated components from several well-established programs, including the Princess Margaret Cancer Centre Prostate Cancer Rehabilitation Clinic (PM-PCRC), and led to the development of the True North Sexual Health and Rehabilitation eClinic (SHAReClinic).

SHAReClinic is a virtual, biopsychosocial program that supports PCa survivors and their partners in managing the physical, emotional, and relational aspects of sexual health after treatment [13]. Unlike traditional in-person clinics, SHAReClinic removes geographic and logistical barriers by providing patients with flexible, expert-led support from the comfort of their own homes. E-visits are conducted by trained sexual health professionals at key recovery milestones, and participants engage independently with tailored educational modules and interactive multimedia content [13]. The program combines biomedical and psychosocial approaches to address topics such as erectile function, sexual performance anxiety, intimacy, and partner concerns. Participants receive guidance on the use of pro-erectile therapies and devices, alongside access to a curated health library, goal-setting tools, and progress trackers that support engagement and self-management [13]. This comprehensive model offers a patient-centred approach to sexual rehabilitation that meets the diverse and evolving needs of individuals and intimate partners following PCa treatment.

To ensure that health care providers (HCPs) are equipped to deliver SHAReClinic effectively, the program incorporates SHAReTraining, an expert consensus online course developed by a multidisciplinary team of psychologists, nurses, researchers, and uro-oncologists [14]. SHAReTraining is designed to prepare HCPs to provide high-quality, PCa-specific sexual health counseling using the same biopsychosocial framework that underpins SHAReClinic. Delivered over 12 weeks, the facilitator-led course avoids a traditional didactic format, instead emphasizing interactive, discussion-based learning through live virtual seminars and structured forums. The curriculum includes topics ranging from sexual health theory and assessment models to erectile rehabilitation, intimacy, and communication strategies with diverse patient populations. Outcomes from real-world evaluations indicate that SHAReTraining improves provider knowledge, confidence, and readiness to initiate sexual health discussions [14]. Together, SHAReClinic and SHAReTraining are an integrated, scalable model of care that leverages digital health technology to enhance access to specialized, evidence-informed sexual health support.

Despite the strength of SHAReClinic and SHAReTraining as a combined intervention and training model, translating innovation into routine clinical practice requires deliberate implementation planning. HCPs often encounter systematic and contextual challenges that impede the real-world application of evidence-based approaches. A pre-implementation assessment can ensure intervention deliverers are better prepared, with targeted strategies to promote success.

### 1.3. Consolidated Framework for Implementation Research (CFIR) 2.0

The Consolidated Framework for Implementation Research (CFIR) 2.0 is comprehensive framework designed to systematically identify factors influencing the implementation of health care innovations across diverse settings [15]. CFIR 2.0 categorizes determinants into five domains: innovation, outer setting, inner setting, individuals, and implementation process, each further subdivided into detailed constructs. Overall, this provides a systematic and detailed approach to explore the many factors that affect implementation in an organized way. Building upon the original CFIR framework, CFIR 2.0 enhances usability by clarifying construct definitions and emphasizing contextual dynamics and equity considerations. CFIR 2.0 is regularly utilized in health care settings to support the identification of specific barriers and facilitators, allowing for the development of tailored strategies to optimize the adoption and sustainability of interventions [16].

### 1.4. Aims and Objectives

The objective of this study is to identify potential barriers and facilitators to the implementation of SHAReClinic across nine Canadian health care centres through a pre-implementation assessment. Guided by the CFIR 2.0, the study aims to identify contextual factors that may impact the adoption, delivery, and sustainability of the intervention. Findings will inform the development of tailored implementation strategies, guide refinements to program design, and support strategic resource allocation to optimize SHAReClinic’s integration.

## 2. Materials and Methods

### 2.1. Study Design

A qualitative descriptive design was used to examine barriers and facilitators influencing the implementation of the SHAReClinic. This approach was chosen due to the topic being largely unexplored and benefiting from a participant-centred account that remains close to the data [17]. Qualitative descriptive design is commonly used in applied health research where practical insights are needed to inform intervention development or implementation strategies [18]. The project was submitted to the University Health Network (UHN) Quality Improvement Review Committee (QIRC) on 6 September 2024 and received approval on 18 September 2024 (QIRC ID# 24-0922). As this study was deemed a quality improvement initiative, QIRC approval waived the requirement for Research Ethics Board approval.

### 2.2. Setting and Participants

This study was initiated at Princess Margaret Cancer Centre (PM) and includes the participation of nine cancer care sites across Canada: Hudson Regional Cancer Centre (formerly Simcoe Muskoka Regional Cancer Program), Ottawa Hospital Cancer Centre, Kingston Health Sciences Centre, London Health Sciences Centre, Trillium Health Partners, Saskatchewan Health Authority, Health Sciences Centre Winnipeg, Alberta Urology Centre, and Nova Scotia Health Authority. Sites were strategically selected to support a nationwide implementation effort, with the aim of achieving broad geographic representation and capturing diversity in institutional size, clinical practices, and patient populations. Particular attention was given to including centres that serve rural and remote communities, where access to specialized sexual health services is often severely limited [7].

Purposive sampling was used to recruit site champions from each participating site to participate in this study. Site champions were selected based on their roles in local leadership or clinical coordination and were identified through existing clinical and research partnerships. Each site champion was invited to participate in a semi-structured interview and invited to identify an additional key knowledge user at their site, such as a future sexual health coach or clinic representative, to join. This approach was intended to capture site-specific knowledge and a range of perspectives on implementation determinants across diverse oncology care settings.

### 2.3. Data Collection

Data were collected by two research analysts (T.I., D.P.) through semi-structured interviews conducted virtually via Microsoft (MS) Teams (version 25163.3001.3726.6503, Microsoft Corporation, Redmond, WA, USA) between 14 November 2024 and 13 February 2025. Each interview lasted 45–60 min and participants provided informed verbal consent prior to beginning.

A semi-structured interview guide was developed using the CFIR 2.0 framework and informed by PM’s internal quality improvement template to ensure all relevant implementation domains were explored systematically [12]. The interview guide can be found in Supplementary Materials (Supplementary File S1). Interview questions explicitly mapped CFIR 2.0 constructs. For example, the following question was used to assess the CFIR 2.0 construct of Inner Setting > Culture: “Can you describe the current culture and practices related to sexual health care for prostate cancer patients and their partners in your institution?”

Interviews were divided into two sections. The first section included background questions to gather contextual information about the participants; roles, site characteristics, and existing sexual health practices. Participants then viewed a five-minute video providing an overview of SHAReClinic, including a walk-through of the platform. The second section focused primarily on perceived enablers and barriers to integrating SHAReClinic into clinical workflows, drawing on participants’ insights related to institutional readiness, resource availability, and potential barriers and facilitators for implementation.

All interviews were audio-recorded, transcribed verbatim, and anonymized to maintain participant confidentiality.

### 2.4. Data Analysis

A qualitative thematic analysis was conducted using a hybrid deductive and inductive approach. The analysis was informed by CFIR 2.0 and followed the principles of applied thematic analysis [19]. This approach was selected given its flexibility, structured coding process, and suitability for framework-guided implementation research [19,20]. Data were managed and analyzed using Dedoose (Version 10.0.23; SocioCultural Research Consultants, LLC, 2025), a cloud-based application for qualitative and mixed methods research [21].

All audio recordings were reviewed prior to and after transcription, and transcripts were re-read to ensure familiarity with the data. To ensure a structured and context-specific approach to coding, each CFIR 2.0 domain was operationalized in advance to reflect its relevance to SHAReClinic implementation. This process established a clear framework for organizing data while allowing space for emerging insights. Table 1 outlines how CFIR 2.0 domains were defined within this study. After defining the domains, the CFIR 2.0 codebook template, including pre-populated definitions of each construct and coding guidelines, was used to facilitate the analysis in Dedoose. To ensure quality and reliability in the coding, two researchers (T.I., D.P.) independently coded the first two randomly selected interviews. Both coders then met with a third team member (S.E.N.-S.), a knowledge translation expert, to review and ensure proper application of CFIR 2.0. The remaining transcripts were coded by one researcher (T.I.) and subsequently reviewed by the second coder (D.P.).

Following the initial coding, all excerpts within each CFIR 2.0 construct were reviewed again by both coders to ensure consistency. Relevant CFIR 2.0 domains and constructs were refined through discussion and applied inductively to the dataset. Descriptions of each domain and subconstruct were developed, and representative quotes were selected for each construct. These can be found in Appendix A, Table A1.

## 3. Results

### 3.1. Participant Characteristics

A total of eight interviews were conducted with 17 participants. Each interview included between one and four participants, with an average of two participants per site. One site declined to participate due to limited time and resources and concerns about the feasibility of implementing SHAReClinic at their site.

Participants included 13 HCPs and four individuals in leadership roles. Provider roles included urologists, general practitioners, nurse practitioners, physician assistants, registered nurses, and a social worker. Leadership participants held positions in quality improvement and program management.

### 3.2. Determinants of Possible Implementation Success

#### 3.2.1. Innovation

Relative Advantage: Participants viewed SHAReClinic’s integration of psychosocial care throughout the intervention as a key advantage over current models, which primarily address physical side effects while overlooking emotional and relational aspects of sexual health. One provider explained, “The oncologist does disclose some of the potential side effects of treatment…But it’s discussed mostly on a medical level, nothing to do with the sexual counseling side of things.” Participants also emphasized that SHAReClinic provides clearer pathways for patient support, filling a gap in current services. One provider noted, “We think SHAReClinic is going to be tremendously helpful…. Right now, we don’t have a clear path as to how to assist these patients and there is a huge amount of need.”

Adaptability: SHAReClinic’s flexibility to be tailored to local needs and workflows was perceived as a strength. Participants appreciated that the online platform could be refined in response to user and site-specific feedback. Some participants discussed modifications to demographic and screening questions to ensure inclusivity. One provider said, “I would prefer, “Do you have a sexual partner?” Because people can be single, but still sexually active.” Participants also noted operational considerations, including the need for system features that would accommodate health coach absences.

Design: While some participants raised concerns about Internet access in remote regions and technology use among older patients, many emphasized SHAReClinic’s accessibility and ease of use. One provider noted, “Our region is large and very remote; Internet connection is a challenge in a lot of our First Nation communities,” while another provider shared, “Some patients are older, so technology is a challenge.”

Despite these concerns, the platform’s design was praised as user-friendly and customizable. One provider called it “a very elegant platform,” and others appreciated features like tailored educational content and self-monitoring tools. As one noted, “I like that they can have trackers… I think they would love it.” The virtual format was also seen as key to increasing access among men who may be reluctant to attend in-person services: “Even without a financial barrier, it’s like pulling teeth to get men to show up for sexual health counselling. Having something they can do privately at home is going to be key.”

#### 3.2.2. Outer Setting

Local Attitudes: Sociocultural beliefs and stigma around sexual health were identified as influential in shaping patients’ willingness to engage with SHAReClinic. Some participants noted that patients often feel embarrassed to seek help, with one stating, “Guys are very embarrassed to talk about this. Getting over that embarrassment is a big deal and a rate limiting step.” Providers also acknowledged hesitancy in initiating conversations about sexual health, which may reflect broader cultural norms as well as individual-level discomfort or uncertainty about how to integrate it into care. One provider explained, “There is still some hesitancy to open the conversation about sexual health with patients…culturally, it’s not as widely accepted and practiced yet.”

Financing: A lack of dedicated funding for sexual health services was widely cited as a barrier to implementing SHAReClinic. Participants emphasized that this challenge reflects broader gaps in survivorship care, particularly for services related to intimacy and sexual well-being. One provider stated, “There is little to no financial support for this survivorship care…it’s not as well-supported as it should be.” While SHAReClinic was seen as a cost-effective alternative to establishing new in-person services, participants emphasized that institutional investment remains essential to support staffing and long-term integration.

#### 3.2.3. Inner Setting

Relational Connections: Interdepartmental collaboration was described as an important contextual factor that could influence implementation. While established relationships were seen as supportive of seamless care and referrals, fragmentation between services raised concerns about coordination and communication during SHAReClinic rollout. Participants noted, “There’s been a real disconnect, even with the nurse in the urology clinic who is completing the same training as our nurse…there’s no connection between them.” Conversely, other sites noted existing factors that support continuity of care. One provider explained, “We are the only center [in this region] that performs radical prostatectomies anymore…so we don’t have a fragmented system.”

Tension for Change: The need for structured sexual health services was widely recognized, with participants pointing to clear gaps in care that SHAReClinic could help address. One provider explained, “We do not [have a standardized practice for sexual health care]…Every provider does this individually. Some patients get great care, and some don’t.” Another provider emphasized the lack of follow-up, stating, “Patients who don’t ask for help, don’t get it …we don’t have standardized follow-up.” Lastly, participants described that clinical demands sometimes overshadow sexual health needs: “We treat [prostate cancer] effectively, but we don’t always address the other aspects of care…We’re trying to keep up with treating the disease, but in doing so, we sometimes miss the patient.”

Compatibility: Participants highlighted both challenges and opportunities related to SHAReClinic’s alignment with existing workflows. Some participants raised concerns about workflow integration, particularly around documentation requirements. For example, in settings where institutional policies require documentation in both SHAReClinic and their hospital’s electronic medical records (EMRs), this was seen as a burden. One participant explained, “One complication is duplicate documentation…our social worker is legally required to document in our hospital’s EMR, as well as in SHAReClinic.” However, others felt SHAReClinic could easily be incorporated into routine care with minimal disruption. As one provider described, “It would be easy to roll out because it’s already based in our system. It’s just one more thing we would add on.”

Relative Priority: Competing institutional initiatives, including a pan-oncology program and transition to a new hospital EMR system, were identified as barriers. One provider said, “Implementation has been stagnant here for the last three to four months because our institution is implementing another pan-oncology program.” Another provider highlighted the strain of concurrent rollouts: “We need the dust to settle first, people’s capacity for change management and information processing needs time.”

Available Resources: While SHAReClinic provides standardized training for sexual health coaches and digital infrastructure, ongoing staffing costs and integration into local workflows remain the responsibility of individual clinics. This shaped perceptions of feasibility across sites, particularly in relation to available personnel and clinical capacity.

The availability of resources to implement SHAReClinic varied widely, with many participants identifying significant barriers related to staffing, funding, and workload. Participants described working in resource-constrained environments where staff were already at full capacity. One provider stated, “Our nursing resources are quite finite…even getting people from diagnosis to treatment. It’s too much.” Another provider emphasized, “All of our resources are fully tapped out right now.” Limited financial support further compounded these challenges. One provider remarked, “The hospital couldn’t have cared less about helping us fund a nurse for the sexual health clinic.”

Some sites reported having infrastructure in place to support SHAReClinic, including trained staff, clinical space, and time for patient care. “We have one counselor who’s already trained and another starting training,” one provider shared. Another provider noted, “[Our nurse] spends about an hour with each patient for a full review of their sexual health.” Many also reported sufficient technological capacity: “We have access to the technology needed to facilitate this.”

#### 3.2.4. Individuals

High-Level Leaders: Lack of motivation from institutional leadership in supporting sexual health services was seen as a barrier to implementation. Participants emphasized the need for senior leadership buy-in to allocate resources and integrate SHAReClinic into routine care. One provider noted, “I need the senior leadership to believe this is necessary and support psycho-oncology.”

Mid-Level Leaders: Participants cited high motivation, but barriers related to the opportunity and capabilities of mid-level leaders, including physicians and referring providers. Participants cited time constraints and awareness gaps as key barriers: “We have a passion. We want to help. We’d love to see patients do better, but you can only do so much.” Others highlighted gaps in knowledge, stating, “It’s a lack of awareness about sexual dysfunction rather than a lack of comfort…people don’t realize it’s an issue, and don’t know where to refer patients.” At the same time, many participants described motivation and support for sexual health care among mid-level leaders. One provider stated, “We have really incredible support and buy-in from physicians, nurses, and radiation therapists for the sexual health clinic.”

Innovation Deliverers: Future sexual health coaches expressed high motivation and enthusiasm, though some also raised concerns about their preparedness to deliver the intervention. Some participants expressed concerns about training adequacy and confidence in delivering care. One provider stated, “Just taking the course… doesn’t feel like enough for someone without a degree in social work or psychology …there has to be some sort of clinical mentorship.” Despite these concerns, participants described growing confidence and motivation. One provider shared, “I’ve learned a lot from the course…now we just need to come up with a game plan.” Others highlighted how training had already led to increased service delivery, “Our social worker has completed the training, she is already seeing patients for sexual health.”

Innovation Recipients: A substantial need for structured sexual health support was widely recognized. One provider explained, “SHAReClinic is definitely needed…a lot of patients say they don’t feel very prepared going into treatment.” The prevalence of sexual health concerns was also noted, with another provider explaining, “A significant number of our patients are reporting moderate to severe issues with sexual health and intimacy.” The importance of setting realistic expectations for patients was also highlighted, as one provider explained, “Patients feel like expectations weren’t set realistically. They go through treatment, have significant side effects, and then they’re left reeling.”

#### 3.2.5. Implementation Process

Assessing Context: Participants identified both barriers and facilitators in preparing for SHAReClinic’s implementation. A primary concern was workload distribution and role overlap, with one provider asking, “For every patient referred, how much of a time commitment per week is that?” Another questioned, “I’m the sexual health coach, consulting physician, and prescriber. Am I referring to myself?” On the other hand, some sites felt confident in navigating implementation, stating, “There are barriers I foresee, but nothing major for us.”

Engaging: Participants identified several strategies to increase awareness and uptake of SHAReClinic among both providers and patients. Grand rounds and structured presentations were suggested as effective ways to engage clinical teams. One provider stated, “Introducing it at a grand round would be probably the best bet,” while another noted, “A quick presentation explaining this is a well-researched initiative would go a long way.” Participants also had ideas for engaging patients, suggesting, “A business card with a QR code would be the best way…people can register on their phone. The easier it is, the more likely they are to do it.” Another participant added, “Pamphlets or similar materials would be excellent.”

Planning: Planning was identified as a facilitator for SHAReClinic’s implementation, with participants outlining clear goals and success criteria. A common objective was ensuring all patients receive sexual health support early in their treatment, with one provider stating, “Our goal is to see all patients, pre-op and pre-radiation.” Others emphasized the need for routine integration into clinical workflows, with one describing success as, “a well-oiled machine, fully embedded in practice.” The importance of seeing structured follow-up for patients was highlighted, with another stating, “I would like proper follow-up after prostate cancer treatment…dealing with it right from the start.”

## 4. Discussion

### 4.1. Interpretation of Findings

This study identified key barriers and facilitators influencing the implementation of SHAReClinic, mapped across the CFIR 2.0 domains. Findings were drawn from interviews conducted across multiple sites in Canada, providing a broad perspective on the factors shaping implementation in diverse clinical settings. The results highlight both the opportunities and challenges associated with integrating a virtual, biopsychosocial sexual health intervention into routine oncology care.

A clear need for SHAReClinic was identified, with participants emphasizing the lack of standardized sexual health support in oncology settings. While some sites had existing services, these were often limited in scope, and most had no formal pathways for addressing sexual health. In addition, many reported a persistent gap in care, stating that patients often feel underinformed and ill prepared for treatment-related sexual dysfunction. This highlights a broader gap in oncology, where sexual health remains under-addressed despite its known impact on quality of life [7,22,23,24].

One potential barrier to implementation is the varying levels of provider knowledge regarding sexual health concerns in oncology, which may impact their ability to navigate these conversations and generate referrals to SHAReClinic. Research suggests that discomfort in discussing sexual health is often rooted in insufficient training rather than a lack of willingness [25,26,27,28,29]. Although SHAReTraining was developed to equip health care providers with the necessary skills, a small number of participants suggested ongoing mentorship or follow-up support could further enhance provider confidence. While this was not a common concern, it may indicate the potential value of regular graduate check-ins or the development of a community to support continued learning, peer connection, and mentorship among SHAReTraining alumni.

SHAReClinic’s virtual design was widely seen as a facilitator, with participants recognizing its potential to increase accessibility. Digital health interventions have been shown to improve engagement and overcome geographical barriers, particularly for patients in remote areas where in-person services are limited [30,31]. While some concerns were raised about older patients’ ability to navigate the technology, participants acknowledged that digital health platforms are becoming increasingly familiar to many patients, and the flexibility of an at-home program may enhance engagement. Virtual access enables patients to participate in care privately and at their own pace, which has been shown to be particularly beneficial when discussing sensitive topics like sexual health [32].

Despite provider motivation and demonstrated patient need, a critical barrier to implementation was the lack of institutional funding and high-level leadership support. Participants described challenges in securing financial resources for sexual health programming, with many noting that survivorship care often falls outside of traditional funding structures. Previous studies have shown that financial constraints and lack of institutional buy-in are common barriers to implementing psychosocial interventions, with most challenges occurring at the organizational level [33,34]. The absence of dedicated funding and leadership advocacy poses a significant risk to SHAReClinic’s long-term viability. Notably, the only site unable to participate in interviews cited time and resource constraints, highlighting the challenges of implementing new programming in an already strained health care system. Moving forward, securing institutional support and embedding SHAReClinic within existing clinical workflows will be critical for its long-term sustainability.

### 4.2. Practice Implications and Actionable Recommendations

To support future implementation planning, we developed a set of action-oriented recommendations informed by CFIR 2.0 constructs and organized using the Expert Recommendations for Implementing Change (ERIC) [35,36]. ERIC provides a standardized nomenclature for implementation strategies, grouped into conceptually distinct clusters to guide selection based on contextual fit and implementation phase [35]. By aligning recommendations with both CFIR 2.0 and ERIC, we aimed to ensure theoretical relevance while offering practical, context-sensitive strategies to support the feasibility, uptake, and long-term sustainability of SHAReClinic. Table 2 presents these key considerations organized by ERIC cluster.

The takeaways presented in Table 2 reflect current insights from the pre-implementation phase and serve as a foundation for planning SHAReClinic rollout across diverse oncology settings. Our team will also be conducting post-implementation interviews to identify emerging challenges, adaptations, and successful strategies. Findings from both phases will inform the development of a comprehensive SHAReClinic Implementation Handbook to support future scale-up and sustainable integration into practice.

### 4.3. Strengths and Limitations

A key strength of this study is its use of the CFIR 2.0 framework, which allowed for a comprehensive and structured evaluation of factors influencing SHAReClinic’s implementation. Additionally, the inclusion of multiple cancer care sites across Canada provides a broad and diverse perspective, enhancing the generalizability of findings. The study also benefits from qualitative insights drawn from a range of knowledge users, including physicians, site administrators, and future sexual health coaches, capturing varied perspectives on implementation facilitators and barriers.

Participation was limited by time and resource constraints, with one site unable to take part, potentially reducing the breadth of perspectives from a highly resource-strained setting. Additionally, while interviews included a diverse range of providers, patient perspectives were not directly captured at this stage. This limits our understanding of patient-specific barriers and facilitators to implementation. However, these perspectives will be explored in the next phase of research through post-implementation interviews with patients to further refine SHAReClinic and address emerging challenges.

## 5. Conclusions

This study highlights both the need for and challenges in implementing SHAReClinic as a structured sexual health intervention for PCa patients. The findings emphasize the importance of institutional support, financial investment, and comprehensive training to ensure successful integration. While SHAReClinic was widely recognized as addressing critical gaps in sexual health care, overcoming systemic barriers such as competing priorities and workflow integration will be essential.

To guide implementation, we identified a series of action-oriented takeaways aligned with CFIR 2.0 constructs, offering guidance for tailoring SHAReClinic to diverse oncology settings. These insights provide a foundation for implementation planning and will continue to evolve through subsequent phases of this work. Moving forward, we will conduct post-implementation interviews to capture real-world adaptations and lessons learned across participating sites. These findings will inform the development of a comprehensive SHAReClinic Implementation Handbook, designed to support long-term sustainability and scale-up across cancer care systems.

## Figures and Tables

**Table 1:** Operationalization of CFIR 2.0 domains in the context of SHAReClinic.

CFIR 2.0 Domain	Operationalization for SHAReClinic Pre-Implementation
Innovation	Refers to SHAReClinic itself, including its overall purpose, structure, and delivery format.
Outer Setting	Encompasses factors outside the hospital system, including broader societal attitudes, financial considerations, and external policies that may influence SHAReClinic’s implementation.
Inner Setting	Focuses on the specific clinical sites where SHAReClinic is being introduced, including local infrastructure, resources, workflows, and interdepartmental relationships.
Individuals	Includes different stakeholders involved in SHAReClinic’s implementation: - High-Level Leaders: Hospital executives, and institutional decision-makers who influence program adoption. - Mid-Level Leaders: Physicians and referring providers responsible for integrating SHAReClinic into patient care. - Innovation Deliverers: Sexual health coaches responsible for delivering SHAReClinic services. - Innovation Recipients: Patients and their partners engaging with SHAReClinic.
Implementation Process	Refers to planning and preparation for SHAReClinic’s launch, as implementation has not yet occurred.

**Table 2:** Implementation strategies for SHAReClinic mapped to ERIC and CFIR 2.0.

ERIC Cluster	ERIC Strategy	CFIR Domain/Construct	Action-Oriented Takeaway
Adapt and tailor to context	Promote adaptability	Inner Setting (Compatibility)	Adjust documentation and integration into EMR systems to reduce duplication and improve alignment with site workflows.
	Tailor strategies	Innovation (Adaptability)	Gather feedback to tailor SHAReClinic to local workflows and patient needs.
Develop stakeholder interrelationships	Build a coalition	Inner Setting (Relational Connections)	Foster interdepartmental collaboration through shared training and communication channels.
	Involve executive boards	Individuals (High-Level Leaders)	Engage senior leadership early to align SHAReClinic with organizational goals and secure buy-in.
Provide interactive assistance	Provide local technical assistance	Innovation (Design)	Address tech-related barriers through patient support and infrastructure accommodations (e.g., remote Internet access).
Train and educate stakeholders	Conduct ongoing training	Individuals (Mid-Level Leaders)	Offer targeted training and resource guides to mid-level leaders to increase awareness and capacity for referral.
	Provide ongoing consultation	Individuals (Innovation Deliverers)	Provide mentorship opportunities and peer support for new sexual health coaches post-training.
	Develop educational materials	Individuals (Mid-Level Leaders), Implementation Process (Engaging), Outer Setting (Local Attitudes)	Deliver targeted training and resource guides to support referrals.
Use evaluative and iterative strategies	Assess for readiness and identify barriers and facilitators	Inner Setting (Available Resources)	Conduct readiness assessments to identify and plan for infrastructure and staffing limitations.
	Develop a formal implementation blueprint	Inner Setting (Tension for Change), Individuals (Innovation Recipients), Implementation Process (Assessing Context, Planning)	Leverage documented care gaps, clarify site roles, and build concrete referral and follow-up plans for routine SHAReClinic integration.
	Stage implementation scale-up	Inner Setting (Relative Priority)	Time SHAReClinic rollout in coordination with other institutional initiatives to prevent change fatigue.
Utilize financial strategies	Access new funding	Outer Setting (Financing), Inner Setting (Available Resources)	Secure sustainable funding to support long-term delivery of SHAReClinic, including early identification and resourcing of key personnel such as sexual health coaches.

## Data Availability

The data presented in this study are available on request from the corresponding author due to privacy and ethical restrictions.

## Supplementary Materials

The following supporting information can be downloaded at: https://www.mdpi.com/article/10.3390/curroncol32070395/s1. Interview Guide.

## Abbreviations

The following abbreviations are used in this manuscript:

CFIR	Consolidated Framework for Implementation Research
EMRs	Electronic medical records
ERIC	Expert Recommendations for Implementing Change
HCP	Health care provider
PCa	Prostate cancer
PM	Princess Margaret Cancer Centre
PM-PCRC	Princess Margaret Cancer Centre Prostate Cancer Rehabilitation Clinic
QIRC	Quality Improvement Review Committee
SD	Sexual dysfunction
SHAReClinic	Sexual Health and Rehabilitation eClinic
UHN	University Health Network

## Appendix A

**Table A1:** CFIR 2.0 domains and constructs with descriptions and representative quotes.

CFIR Construct	Description	Barrier or Facilitator	Examples
INNOVATION			
Relative advantage	The degree to which SHAReClinic offers advantages over current practice by addressing gaps in care and providing additional resources.	Facilitator	“The oncologist does disclose some of the potential side effects of treatment…But it’s discussed mostly on a medical level, nothing to do with the sexual counseling side of things.” “We don’t have a process in place right now, so SHAReClinic is better than nothing…[our] survivorship clinic will offer similar basic education, but not all the resources that SHAReClinic has.” “We think SHAReClinic is going to be tremendously helpful…. Right now, we don’t have a clear path as to how to assist these patients and there is a huge amount of need.” “We do the biology. But nobody talks about, how are you feeling about this? Your intimacy? Your relationships? Yourself? We just don’t do it.”
Adaptability	The degree to which SHAReClinic can be modified, tailored, or refined to fit local context or needs.	Facilitator	“When our sexual health coach is away, does the SHAReClinic have an “out of office”?” “When it asked about sexual orientation… is there a full spectrum they can choose from?” “I would prefer, “Do you have a sexual partner?” Because people can be single, but still sexually active.”
Design	The degree to which SHAReClinic is well designed and packaged.	Barrier	“Our region is large and very remote; internet connection is a challenge in a lot of First Nation communities.” “The platform may be tricky, but we’ll see. I’m sure patients could figure it out. People have become more adept to the internet.” “Some patients are older, so technology is a challenge.”
		Facilitator	“It’s a very elegant platform and has a lot of potential. I like that we could pick pieces of the library for patients…I think it would be remarkable.” “I think it seems very good and patient friendly.” “I like that they can do it in the comfort of their own space…They don’t feel intimidated having to come here and talk about their most intimate concerns.” “I like the that they can have trackers…I think they would love it.” “Even without a financial barrier, it’s like pulling teeth to get men to show up for sexual health counselling. Having something they can do privately at home is going to be key,”
OUTER SETTING			
Local attitudes	The degree to which sociocultural values and beliefs encourage support for sexual health care and the implementation of SHAReClinic.	Barrier	“Guys are very embarrassed to talk about this. Getting over that embarrassment is a big deal and a rate limiting step.” “There is still some hesitancy to open the conversation about sexual health with patients…culturally, it’s not as widely accepted and practiced yet.”
Financing	The degree to which external funding is available to support implementation.	Barrier	“Our province didn’t cover penile prostheses for 25 years. We spent over eight years trying to get it approved.” “There is little to no financial support for this survivorship care…it’s not as well-supported as it should be.” “There’s no grant that’s really out there that’s wanting to fund an education position [for sexual health care].”
INNER SETTING			
Relational connections	The degree to which there are high-quality formal and informal relationships, networks, and teams within and across Inner Setting boundaries.	Barrier	“There’s been a real disconnect, even with the nurse in the urology clinic who is completing the same training as our nurse…there’s no connection between them.” “Our urology clinic doesn’t have much insight into the current Cancer Centre…it’s quite fragmented.”
		Facilitator	“We are the only center [in this region] that performs radical prostatectomies anymore…so we don’t have a fragmented system.” “The hospital’s Indigenous Patient Services have really good relationships with health directors throughout the region.”
Tension for change	The degree to which the current situation is intolerable and needs to change.	Facilitator	“We do not [have a standardized practice for sexual health care]…Every provider does this individually. Some patients get great care, and some don’t.” “Huge. There’s a huge demand [for sexual health services].” “Patients who don’t ask for help, don’t get it …we don’t have standardized follow-up.” “We need a better system to reduce the barriers patients face.” “We treat [prostate cancer] effectively, but we don’t always address the other aspects of care…We’re trying to keep up with treating the disease, but in doing so, we sometimes miss the patient.”
Compatibility	The degree to which the innovation fits with workflows, systems, and processes.	Barrier	“One complication is duplicate documentation…our social worker is legally required to document in our hospital’s EMR, as well as in SHAReClinic.” “Physicians are already in so many different systems, I don’t know how well utilized it would be on their end.”
		Facilitator	“It would be easy to roll out because it’s already based in our system. It’s just one more thing we would add on.’”
Relative priority	The degree to which implementing and delivering the innovation is important compared with other initiatives.	Barrier	“Implementation has been stagnant here for the last three to four months because our institution is implementing another pan-oncology program.” “This is the most extended I’ve ever seen the health care system. Our physicians aren’t immune-everyone is having to prioritize their time and attention.” “We’re going live with a regional health information system that’s changing the way everyone works… We need the dust to settle first, people’s capacity for change management and information processing needs time.”
Available resources	The degree to which resources (staff, space, technology) are available to implement and deliver the innovation.	Barrier	“Ideally, the nurse tied to our prostate pod would fill this role, but we don’t have anyone right now.” “We don’t have a sexual health coach or funding for one…it would be impossible to do this without that.” “All of our, resources are fully tapped out right now.” “Our nursing resources are quite finite… even getting people from diagnosis to treatment. It’s too much.” “The hospital couldn’t have cared less about helping us fund a nurse for the sexual health clinic.” “Physical space is always at a premium.”
		Facilitator	“We have one counselor already trained and another starting training.” “We have tons of space in our facility right now.” “[Our nurse] spends about an hour with each patient for a full review of their sexual health.” “We have access to the technology needed to facilitate this.”
INDIVIDUALS			
High-level leaders (CEOs, government, hospital heads)	The degree to which high-level leaders (ex. hospital CEOs) have the need, capability, opportunity and motivation to support SHAReClinic.	Barrier	“We just need institutional buy in.” “I need senior leadership to believe that this is necessary, and support psycho-oncology.”
Mid-level leaders (managers, physicians, frontline referring to SHARe)	The degree to which mid-level leaders (Ex. referring physicians, site managers) have the need, capability, opportunity and motivation to support SHAReClinic.	Barrier	“We have a passion. We want to help. We’d love to see patients do better, but you can only do so much.” “It’s a lack of awareness about sexual dysfunction rather than a lack of comfort…people don’t realize it’s an issue, and don’t know where to refer patients.” “As radiation therapists, we weren’t taught to discuss this [sexual health] with patients.”
		Facilitator	“We have really incredible support and buy in from physicians, nurses. and radiation therapists for the sexual health clinic.” “We have a lot of internal support from all interdisciplinary teams.”
Innovation deliverers (health care providers working in SHARe—also known as coaches)	The degree to which innovation deliverers (ex. sexual health coaches) have the need, capability, opportunity, and motivation to support SHAReClinic.	Barrier	“Just taking the course… doesn’t feel like enough for someone without a degree in social work or psychology …there has to be some sort of clinical mentorship.” “I don’t know that we’d be able to properly walk a patient through that [sensate focus]…I wouldn’t have the time or comfort to fully guide a patient through those exercises.”
		Facilitator	“I’ve learned a lot from the course…now we just need to come up with a game plan.” “I had no training in managing male sexual health in oncology…. I was desperate to find resources and that’s how I came across the SHAReClinic training.” “Our social worker has completed the training, she is already seeing patients for sexual health.”
Innovation recipients (patients)	The degree to which innovation recipients (ex. PCa patients and partners) have the need, capability, opportunity, and motivation to support/participate in SHAReClinic.	Facilitator	“SHAReClinic is definitely needed…a lot of patients say they don’t feel very prepared going into treatment.” “We get a lot of questions about injections, the vacuum erection device (VED) and where to find sexual health resources.” “A significant number of our patients are reporting moderate to severe issues with sexual health and intimacy.” “A number of men have concerns about their masculinity or feel emasculated without erectile function.” “Patients feel like expectations weren’t set realistically. They go through treatment, have significant side effects, and then they’re left reeling.” “We know that there is an appetite from patients.”
IMPLEMENTATION PROCESS			
Assessing context	The degree to which the site is identifying and preparing for anticipated challenges and enablers to implementing SHAReClinic.	Barrier	“For every patient referred, how much of a time commitment per week is that?” “I’m the sexual health coach, consulting physician, and prescriber. Am I referring to myself?”
		Facilitator	“There are barriers I foresee, but nothing major for us.” “We’re lucky there’s a lot of latitude in private practice, so I don’t foresee any challenges.” “Planning isn’t just about referrals and bookings…empowering frontline staff is a key piece of our sustainability planning.”
Engaging	The degree to which individuals attract and encourage participation for innovations deliverers to deliver the implementation and for innovation recipients to patriciate/receive the innovation.	Facilitator	“Introducing it at a grand rounds would be probably the best bet.” “A quick presentation explaining this is a well-researched initiative would go a long way.” “Start with an email blast “We have this program…send all pre-treatment patients here” They could distribute it to nurse practitioners and others, with clear explanations of how to refer.” “We should look for real engagement…breakfast sessions, lunch and learns, or a short video staff can watch when they have a minute.” “A business card with a QR code would be the best way…people can register on their phone. The easier it is, the more likely they are to do it.” “Pamphlets or similar materials would be excellent.”
Planning	The degree to which individuals define implementation goals and outline success criteria for SHAReClinic.	Facilitator	“Our goal is to see all patients, pre-op and pre-radiation.” “Success would a well-oiled machine, fully embedded in practice.” “A universal approach- with all of us are referring into the clinic as much as we can.” “I would like to proper follow up after prostate cancer treatment…dealing with it right from the start.”
