# Supplementary material for: Pre-Implementation Assessment of a Sexual Health eClinic in Canadian Oncology Care
Source: Curr Oncol. 2025 Jul 10;32(7):395. doi: 10.3390/curroncol32070395 (PMC12293689; doi:10.3390/curroncol32070395)
Supplement: Supplementary file 1 [file curroncol-32-00395-s001.zip › Interview Guide.pdf]

Implementing SHAReClinic
Interview Guide

https://cfirguide.org/constructs/

Preamble:

Thank you for taking the time to meet with us today. As you are aware, we are embarking on an initiative aimed at addressing sexual dysfunction (SD) in patients who have undergone prostate cancer (PCa) treatment. Given the significant prevalence of SD post-treatment and its impact on both patients and their partners, there is a critical need for accessible, high-quality treatment options.

Our project involves the implementation of the Sexual Health and Rehabilitation eClinic (SHAReClinic), a web-based innovation designed specifically for patients and partners affected by SD following PCa treatment. Building on a successful pilot study, which demonstrated high patient engagement and effective outcomes, our goal is to integrate SHAReClinic into nine Canadian Cancer Centres.

During this interview, I will ask questions about your clinic's current practices, the potential challenges and benefits of incorporating SHAReClinic into your workflow, and your readiness for this implementation. We are particularly interested in your insights into strategies that could facilitate the successful adoption and sustainment of SHAReClinic at your site.

The interview will be approximately 45 minutes and will be audio recorded to accurately capture your responses. Please rest assured that your feedback will remain confidential, and any personal information will be protected. Feel free to skip any questions you are uncomfortable with.

Do you have any questions before we start?
[No] Great. I will begin recording now, if that is alright with you?

Questions

1. Let's start with a brief round of introductions. Please describe your role within your site and within this project.

First, I would like to understand the existing work processes and practices in your setting.

2. Can you describe the current culture and practices related to sexual healthcare for prostate cancer and their partners in your institution? *[Inner Setting > Culture*]
   a. What are the typical processes or referral mechanisms for addressing sexual dysfunction in prostate cancer patients? [*Inner setting >Work Infrastructure*]
   b. Do you believe there would be sufficient demand or interest in expanding services in this area?
      i. Prompt: from patients? From clinicians?
      ii. Would there be any pushback or hesitations? *[Inner Setting > Relative Priority]*

Next, I would like to present a brief overview of SHAReClinic

3. Do you have any questions about SHAReClinic? *[Innovation Domain>Innovation Complexity]*

   a. How useful do you think SHAReClinic would be in your setting?
   b. How complicated or easy does the SHAReClinic seem to use? *[Innovation Domain> Innovation Complexity]*
   c. How does SHAReClinic's goals and benefits compare to existing interventions at your site? What stands out to you in terms of its potential impact on patient care? *[Innovation Domain> Innovation Relative Advantage]*
   d. What features of SHAReClinic would need to be changed or added to better fit your setting? [*Innovation Domain> Innovation Adaptability]*

Next, I would like to understand how the implementation of the system could fit within these existing processes.

4. How well does SHAReClinic fit with existing work processes and practices in your setting? *[Inner Setting > Compatibility and Mission Alignment]*

   a. Will the system replace or complement a current program or process? Are there competing priorities that could affect the implementation of SHAReClinic? *[Inner Setting > Relative Priority]*
   b. What are likely issues or complications that may arise? *[Inner Setting > Compatibility]*

5. What resources (e.g., staff time, financial resources, space, or equipment) are currently available to support SHAReClinic, and what additional resources would be needed? [*Inner Setting > Available Resources]*

   a. What, if any, changes to the infrastructure (e.g. IT systems, physical space) would need to be made to support SHAReClinic? [*Inner setting > Available Resources*]

6. What would be the best way to introduce SHAReClinic to patients? [*Implementation Process > Innovation recipients]*

   a. Who would be the right person/role to introduce and explain SHAReClinic to patients?
      i. Would it be the surgical/medical/radiation oncologists, nurses (which nurse in which role), radiation therapists, and/or administrative staff? [*Individuals Domain > Innovation Deliverers]*
      ii. [Of groups discussed] – do they have the capabilities to deliver this information? Would they be motivated to do so?
      iii. Do they have sufficient capacity to take on this role? Or are there constraints? [*Individuals Domain >Opportunity and Capability]*

7. What kinds of information and materials about the system should be planned for both providers and patients in your setting? *[Inner Setting >Communications]*

   a. What should be the communication or education strategy for getting the word out about SHAReClinic to patients and staff? What materials/modes/venues should be

used? For example, pamphlets, emails, brochures? *[Inner setting > Physical, Information Technology and Work Infrastructure]*

Given everything we've discussed, I'd like to hear your feedback on the implementation of SHAReClinic in your setting.

8. Are there any significant conditions/policies/pressures that you believe would be a barrier to SHAReClinic in your setting? [*Outer Setting > External pressures, local conditions]*

9. What would help get you ready to implement the system? *[Inner Setting >Compatibility]*

10. What would the success of the implementation of SHAReClinic look like to you? [*Inner setting > Mission Alignment]*

Is there anything else that you would like to share related to the implementation of the system?

Thank you for taking the time to participate in this interview.
